# Exercise testing in a paediatric chronic pain cohort – A cross-sectional observation study

**DOI:** 10.1177/20494637261445408

**Published:** 2026-04-29

**Authors:** Andrew Gorrie, Bethany McLennan, Emily Yeoh, Tiina Jaaniste

**Affiliations:** 1 School of Clinical Medicine, UNSW Sydney, Kensington, NSW, Australia; 2 Physiotherapy Department, Sydney Children’s Hospital, Randwick, NSW, Australia; 3 Department of Pain Medicine, Sydney Children’s Hospital, Randwick, NSW, Australia

**Keywords:** paediatric, chronic pain, exercise testing, patient reported outcome measures

## Abstract

**Background:**

The utility of exercise testing in an interdisciplinary paediatric chronic pain assessment is unknown. The aims of this study were to explore exercise testing results in a paediatric chronic pain cohort and associations with baseline patient-reported outcome measures (PROMs), including various measures of pain experience and functioning.

**Methods:**

There were 153 participants, aged 6–17 years, attending the Sydney Children's Hospital Interdisciplinary Chronic and Complex Pain Clinic with data analysed retrospectively. Exercise tests included the six-minute walk test (6MWT), plank, prone extension and pushups. Results were described and stratified by age, and Spearmen correlation coefficients (*r*_s_) were determined between baseline exercise testing results and PROMs, and between different exercise tests.

**Results:**

The 6MWT distances were greater in the younger subgroup, however, there were no other differences in exercise tests between age groups. Associations between baseline exercise testing results and baseline PROMs ranged from very weak to moderate (*r*_s_’s = .18-.40). Associations between different exercise testing results ranged from very weak to moderate (*r*_s_’s = .17-.52). The 6MWT, plank and pushups each had at least five weak to moderate associations with PROMs and the prone extension test had one weak association.

**Conclusions:**

Exercise testing results in a paediatric chronic pain cohort are presented, including with age stratification. The results of the current study suggest that exercise testing provides unique information to supplement other forms of assessment. There is a need for standardised exercise protocols with normative datasets, so that clinical and research findings are more comparable across settings.

## Introduction

Chronic pain refers to persistent or recurrent pain lasting longer than three months.^1^ Paediatric chronic pain has a reported prevalence of 20.8%.^2^ Children and adolescents with chronic pain experience significantly impaired health-related quality of life (QOL),^3^ pain-related disability,^4^ diminished school attendance^5^ and reduced educational attainment.^6^

The complex nature of chronic pain is best understood with a biopsychosocial model.^7^ Interdisciplinary interventions provide an evidence-based approach for paediatric chronic pain management.^8^ The assessing team will usually include a pain specialist, physiotherapist, psychologist, and other health professionals.^9^ A multimodal pain assessment can incorporate age-appropriate health screening questionnaires, interviews concerning biological, psychological and social domains, and standardised assessments of psychological and physical functioning.^7^

The chronic pain assessment typically includes a physical examination of the child by the doctor and physiotherapist.^9^ This includes screening for red flags that indicate a biological cause of the pain requiring further investigation or treatment.^10^ Somatosensory testing is also supported for use as part of the physical examination.^11^ Interdisciplinary paediatric pain interventions focus on improving function,^8^ yet there is no empirical evidence regarding the assessment of physical functioning in children. Several non-validated methods of exercise testing are described in adult chronic pain literature to assess physical function, most notably the six-minute walk test (6MWT).^12,13^ Exercise test results demonstrate functional, submaximal performance,^14^ with performance affected by effort.^13^ The utility of exercise testing in the overall assessment and management of paediatric chronic pain is unknown.

This hypothesis-generating study investigated the relevance and potential benefits of performing exercise testing as part of an initial assessment in a paediatric chronic pain cohort. The first aim was to describe exercise testing results in a paediatric chronic pain cohort, with age stratification to account for potential age-related differences in children’s capacity to engage in exercise testing.^15^ The second aim was to explore associations between baseline exercise testing results and baseline patient-reported outcome measures (PROMs), most notably measures of pain experience and functioning.

## Methods

### Study design and setting

This was a retrospective, hypothesis-generating study utilising medical records and questionnaires administered as part of the Paediatric electronic Persistent Pain Outcomes Collaboration (PaedePPOC) database.^16^ The study took place at the Sydney Children's Hospital Interdisciplinary Chronic and Complex Pain Clinic. The service assesses and treats children and young people, under the age of 18 years, with chronic pain. Data was retrieved for baseline PROMs from questionnaire responses included in the PaedePPOC database^16^ completed prior to initial clinic assessment. The initial clinic assessment includes a biopsychosocial interview, somatosensory testing and physical examination that includes exercise testing.

Ethics approval was obtained from the Sydney Children's Hospitals Network Human Research Ethics Committee (2023/ETH01429, July 24, 2023). Prior to their completion of the PaedePPOC questionnaires, children and families are made aware that their responses will be used for clinical purposes and potentially for approved research purposes. The current study also included a retrospective analysis of records associated with routine exercise testing. In light of the retrospective nature of this study, the need for study-specific informed consent was waived by the Ethics Committee.

### Participants and recruitment

Participants were identified by screening all electronic medical records of clinic attendees with the following inclusion/exclusion criteria. They were included if they: (1) attended the Sydney Children's Hospital Interdisciplinary Chronic and Complex Pain Clinic for initial multidisciplinary, face-to-face assessment between 2017 and 2022, (2) were between six and 18 years of age, (3) had pain duration greater than three months and (4) completed self-assessment PROMs prior to initial assessment (participants who were non-English speaking or had cognitive impairment were not routinely asked to complete the self-reported PROMs). Participants were excluded if: (1) their pain was cancer-related or (2) they did not have exercise testing performed as part of their initial assessment. COVID-19 impacted the study period as most assessments in 2020–2021 occurred by telehealth, with no exercise testing. The study period was pragmatically determined, starting from when exercise testing was routinely performed with this protocol.

**Figure 1.**
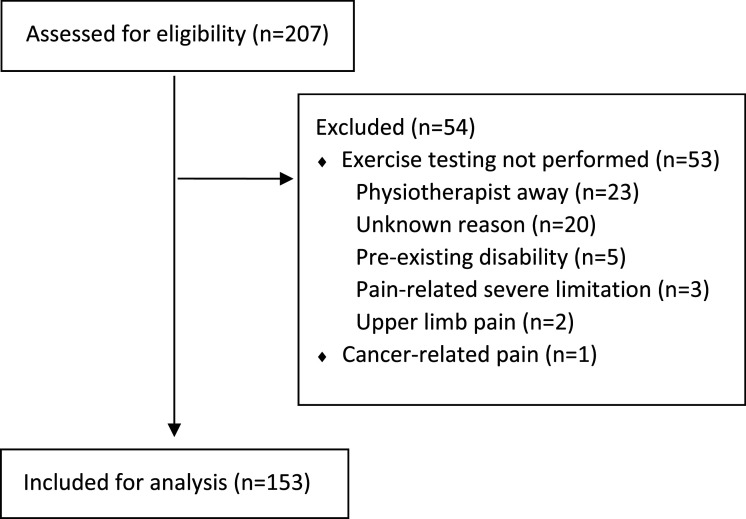
Inclusion/exclusion flow diagram.

There were 207 potential participants identified. The inclusion/exclusion flow diagram is presented in Figure 1. Of the 54 excluded participants, 53 did not have exercise testing performed and one had cancer-related pain. Exercise testing was not performed due to the physiotherapist being away (*n* = 23), unknown/undocumented reasons (*n* = 20), pre-existing physical disability significant enough that they could not attempt the standardised exercise tests (*n* = 5), severe pain-related impairment leading to wheelchair dependence (*n* = 3) and upper limb impairment with a different assessment focus (*n* = 2).

### Study outcomes and measurements

The variables of interest were retrieved from electronic medical records and the PaedePPOC database. Participant demographics included: age, sex, pain duration and pain location. Pain duration was categorised as between three and 12 months, or greater than 12 months. Pain location was categorised as lower limb, upper limb, central or widespread. Height and weight were also included to determine ‘6MWT percent of predicted’.

### Baseline PROMs recorded from PaedePPOC database

#### Pain intensity

Patients are asked to self-report their ‘worst’ and ‘average’ pain over the past week. Children aged 5–7 years used the Faces Pain Scale – Revised,^17^ whereas youth aged 8–18 years used the numeric pain rating from the modified Brief Pain Inventory (BPI). PaedePPOC collects a modified version of the BPI. Youth self-rate their pain intensity from zero to 10. The BPI is modified by rating pain intensity over the past week, instead of the traditional 24 hours. This study used ‘worst pain’ and ‘average pain’ ratings. Although the BPI was developed for adult use,^18^ self-reported numeric rating scales have been widely used for youths with pain.^19,20^

#### Paediatric quality of life inventory (PedsQL^TM^)

The PedsQL^TM^ is a self-reported 23-item tool assessing paediatric health-related quality of life. The items are scored on three-point Likert scale for 5–7 year olds, and five-point scale for 8–18 year olds. There are four subscales (physical, emotional, social and school) which may be grouped into two domains – physical and psychosocial (emotional, social and school). Scores are presented as a percentage, with higher scores reflecting higher quality of life. It is validated for use with paediatric populations.^21^

#### Functional disability inventory (FDI)

The FDI is a self-reported measure of functional impairment. There are 15 items scored on a five-point Likert scale, allowing a maximum total score of 60. Higher scores reflect greater level of disability. Children aged 8–18 years in the PaedePPOC database completed the questionnaire. The FDI is validated in paediatric chronic pain populations in this age group.^22^

#### Bath adolescent pain questionnaire (BAPQ) – Pain-specific anxiety subscale

The PaedePPOC adolescent patient questionnaire includes the seven subscale questions from the BAPQ assessing pain related anxiety. Questions are scored on a five-point Likert scale, for a possible total score of 28, with higher scores reflecting higher pain-related anxiety. Adolescents aged 13–18 in the PaedePPOC database completed the questionnaire. The BAPQ is yet to be validated, but has published data in paediatric pain cohorts.^23^

#### School days missed

The parent/carer is asked the number of school days missed in the past fortnight, for a total possible score of 10. It is not a validated measure.

### Exercise tests

Participants were advised to complete the following exercise tests to maximum effort, self-limited by pain, fatigue and motivation.

#### Six minute walk test (6MWT)

A 6MWT was performed, modified from the American Thoracic Society’s 6MWT protocol for cardiopulmonary assessment to assess functional exercise capacity.^14^ Participants were asked to walk as fast as they can on a 15 m straight track, so that in six minutes they walk as far as they can. Pain and exertion were rated before and after the test. Participants were allowed to take rests as required without the timer pausing, and they were asked to continue walking as soon as they were able. Total distance walked in metres was recorded.

#### Six minute walk test percent of predicted (6MWT % predicted)

The following reference equations by Ulrich et al., (2013) determined a predicted 6MWT for participants based on sex, age, height and weight, in metres.^15^ Reference values were determined from a cohort of children, from five years of age.^15^

Males <13 years: 24.18 x age (years) + 385.18.

Males ≥13 years: 13.08 x age (years) + 476.69.

Females <12 years: 330.07 x height (metres) + 153.3.

Females ≥12 years: −1.867 x weight (kilograms) + 734.29.

A percent of predicted 6MWT (6MWT % Predicted) was determined from the 6MWT raw score and predicted 6MWT.

#### Plank

The plank was performed, with participants instructed to hold a static prone posture, with toes and forearms in contact with the floor. The rest of the body was elevated from the floor, with a neutral spine, creating a straight line from head to ankles. If a participant deviated from the position, they were given one opportunity to correct the position, or the test was stopped. This plank protocol was consistent with a Boyer et al. (2012) study, showing reliability and validity to test muscular endurance of the torso in children as young as eight years.^24^ Our test was ceased if a participant reached 60 s, which differs from the no-time-limit protocol in the Boyer et al. study.^24^

#### Prone extension

The prone extension test was performed with the head, chest, arms and legs elevated from the floor, with the elbows and knees in extension. A study by Lin et al. (2010) showed reliability and validity of a prone extension test in assessing endurance of trunk musculature, proximal extensors and hip abductors, in children as young as four years.^25^ In our study arms were held by the side, instead of in front of the body, due to the personal preference of the assessor. Our test ceased if the participant reached 60 s, as opposed to 120 s in the Lin et al. (2010) study.^25^

#### Pushups in one minute

Patients were instructed to keep hands and toes in contact with the floor, trunk in plank position, feet apart and hands under shoulders. Participants lowered themselves to the floor to form a 90° angle at the elbow. Pushups were performed with similar technique to a study by Henriques-Neto et al. (2020), showing reliability in assessing upper body muscular fitness in children from nine years of age.^26^ Whereas the Henriques-Neto et al. protocol required that pushups were completed every three seconds and counted the maximum achieved,^26^ our participants performed as many pushups as they could in one minute, with as many breaks as required. Where participants felt unable to perform pushups, they were instructed to perform flexed ‘knee pushups’.^27^ The trunk remained straight, but the participants had knees, not toes, in contact with the floor. The two push-up types were analysed separately and as a combined measure (full or knees).

### Statistical analysis

Statistical analysis was completed with software SAS 9.4. For aim one, descriptive statistics were generated from PROMS and exercise testing results. Results were also stratified by age (adolescent and pre-adolescent). Sub-groups were compared using independent samples t-tests for parametric data, and Mann–Whitney U Tests for non-parametric data. Associations between exercise testing, PROMs and age were determined using the Spearman correlation coefficient (*r*_s_). Spearman’s correlation coefficient was chosen due to the non-parametric data distribution. Spearman correlation coefficients were classified as follows: 0–.19 very weak, .20–.39 weak, .40–.59 moderate, .60–.79 strong and .80–1 very strong.^28^

## Results

There were 153 participants included in the analysis. Participant demographics are presented in Table 1. Participants were aged 6–17 years of age, with a median of 14 (interquartile range [IQR] 11–15). PROM and exercise testing results, with age stratification, are presented in Table 2.

**Table 1.** Participant demographics (*N* = 153).

	Frequency	Percent (%)
Female	91	59
Male	62	41
Pain duration		
3–12 Months	54	35
>12 Months	99	65
Pain location		
Lower limb	20	13
Upper limb	7	5
Central	94	61
Widespread	32	21

**Table 2.** Baseline PROMs and exercise testing results, with age stratification.

	Total			Age 6–12 years			Age 13–18 years			
	*N (%)*	Mean *(SD)*	Median *(IQR)*	*N*	Mean (*SD*)	Median (*IQR*)	*N*	Mean (*SD*)	Median (*IQR*)	P
Pain – worst	153 (*100*)		8.0 (*7.0–9.0*)	58		7.5 (*6.3–8.0*)	95		8.0 (*7.0–9.0*)	.32
Pain – average	153 (*100*)		5.0 (*4.0–7.0*)	58		5.0 (*4.0–6.0*)	95		5.0 (*4.0–7.0*)	.56
QOL physical (%)	153 (*100*)		40.6 (*28.1–56.3*)	58		45.3 (*32.7–58.6*)	95		40.6 (*25.0–56.3*)	.13
QOL psychosocial (%)	153 (*100*)	54.0 (*18.1*)		58	55.5 (*16.5*)		95	53.0 (*19.1*)		.42
QOL total (%)	153 (*100*)	50.2 (*17.1*)		58	52.7 (*15.5*)		95	48.7 (*17.8*)		.16
FDI	149 (*97*)	25.2 (*12.2*)		54	23.8 (*11.4*)		95	26.1 (*12.6*)		.27
BAPQ	78 (*51*)	16.4 (*5.5*)		0	-		77	16.5 (*5.4*)		-
School days missed	140 (*92*)		2.0 (*1.0–7.0*)	55		2.0 (*0.5–3.3*)	85		3.0 (*1.0-8.0*)	.01
6MWT (m)	151 (*99*)		495.0 (*430.0–547.5*)	58		517.5 (*450.0–555.0*)	93		487.5 (*420.0–525.0*)	.04
6MWT % predicted (%)	150 (*98*)		78.5 (*67.7–86.3*)	57		82.2 (*71.6–89.5*)	93		76.4 (*66.7–83.2*)	.03
Plank (s)	144 (*94*)		46.0 (*26.5–60.0*)	56		54.0 (*30.0–60.0*)	88		45.0 (*24.8–60.0*)	.42
Prone extension (s)	131 (*86*)		60.0 (*26.0–60.0*)	49		60.0 (*25.0–60.0*)	82		60.0 (*26.0–60.0*)	.72
Pushups – combined	125 (*82*)	18.4 (*8.4*)		58	20.0 (*8.5*)		95	17.3 (*8.2*)		.08
Pushups – full	66 (*43*)	19.1 (*9.1*)		27	20.4 (*8.6*)		39	18.3 (*9.5*)		.36
Pushups – knee	59 (*39*)	17.5 (*7.4*)		23	19.5 (*8.6*)		36	16.2 (*6.3*)		.09

Mean (*SD*) for variables with normative skew, Median (*IQR*) for variables with non-normative skew, P = Independent Samples T-Test for normally distributed variables and Mann–Whitney U Test for non-normally distributed variables.

*SD*, standard deviation; *IQR,* interquartile range; QOL, quality of life; FDI, Functional Disability Inventory; BAPQ, Bath Adolescent Pain Questionnaire Pain-Specific Anxiety Subscale; School Days Missed (in past fortnight); 6MWT, Six Minute Walk Test; 6MWT % Predicted, Six Minute Walk Test Percent of Predicted; Pushups – combined, combined full or knee pushups.

There were two statistically significant differences in baseline exercise testing results between the pre-adolescent and adolescent subgroups (Table 2). The younger group achieved a 6MWT median of 517.5 m (IQR: 450–555) and percent of predicted 6MWT median of 82.2% (IQR: 71.6–89.5), compared to the older group’s 487.5 m (IQR: 420–525) and 76.4% (IQR: 66.7–83.2), respectively. There were no statistically significant correlations between age and exercise testing results (Table 3) or PROMs (Supplemental Table 1), except for very weak correlation to school days missed in the previous fortnight (*r*_s_ = .17). There were no obvious outliers for PROMS or exercise testing results by age on dot plots (Supplemental Figure 1).

**Table 3.** Spearman correlation coefficients (and sample size) between baseline PROMs and exercise testing results.

	6MWT	6MWT % predicted	Plank	Prone extension	Pushups - combined	Pushups - full	Pushups - knee
Pain – worst	−.13 (151)	−.12 (150)	−.22^a^ (144)	−.09 (131)	−.08 (125)	−.19 (66)	.00 (59)
Pain – average	−.07 (151)	−.09 (150)	−.20^a^ (144)	−.13 (131)	−.04 (125)	−.02 (66)	−.03 (59)
QOL physical	.40^aa^ (151)	.38^aa^ (150)	.33^aa^ (144)	.26^a^ (131)	.32^aa^ (125)	.33^a^ (66)	.33^a^ (59)
QOL psychosocial	.22^a^ (151)	.16 (150)	.24^a^ (144)	.10 (131)	.24^a^ (125)	.24 (66)	.24 (59)
QOL total	.30^aa^ (151)	.24^a^ (150)	.29^a^ (144)	.17 (131)	.29^a^ (125)	.29^a^ (66)	.29^a^ (59)
FDI	−.31^aa^ (147)	−.27^a^ (146)	−.26^a^ (140)	−.16 (129)	−.25^a^ (123)	−.22 (65)	−.30^a^ (58)
BAPQ	.00 (77)	.07 (77)	−.24^a^ (71)	−.02 (67)	−.06 (60)	−.05 (29)	−.05 (31)
School days missed	−.20^a^ (138)	−.18^a^ (137)	−.16 (132)	−.07 (122)	−.26^a^ (114)	−.32^a^ (61)	−.19 (53)
Age	−.05 (151)	−.07 (150)	−.02 (144)	.13 (131)	−.16 (125)	−.16 (66)	−.16 (59)

^a^*P* < .05, ^aa^*P* < .001.

QOL, quality of life; FDI, Functional Disability Inventory; BAPQ, Bath Adolescent Pain Questionnaire Pain-Specific Anxiety Subscale; School Days Missed (in past fortnight); 6MWT, Six Minute Walk Test; 6MWT % Predicted, Six Minute Walk Test Percent of Predicted; Pushups – combined, combined full or knee pushups.

The Spearman correlations between all exercise tests and baseline PROMs are shown in Table 3. Spearman correlations between different exercise tests are shown in Table 4. All significant correlations showed higher exercise testing scores were associated with lower PROM impairment, and higher scores on other exercise tests.

**Table 4.** Spearman correlation coefficients (and sample size) between exercise testing results and exercise testing results.

	6MWT	6MWT % predicted	Plank	Prone extension
Plank	.25^a^ (142)	.17^a^ (141)		
Prone extension	.24^a^ (129)	.23^a^ (128)	.52^aa^ (129)	
Pushups - combined	.20^a^ (124)	.19^a^ (123)	.35^aa^ (124)	.24^a^ (117)
Pushups - full	.17 (65)	.15 (64)	.39^a^ (65)	.19 (62)
Pushups - knee	.21 (59)	.21 (59)	.32^a^ (59)	.30^a^ (55)

^a^*P* < .05, ^aa^*P* < .001.

6MWT, Six Minute Walk Test; 6MWT % Predicted, Six Minute Walk Test Percent of Predicted; Pushups – combined, combined full or knee pushups.

The 6MWT had five statistically significant relationships with PROMs. There were four weak associations and one moderate, with *r*_s_’s ranging from −.20 (for school days missed) to .40 (for physical QOL); .40 being the highest correlation between any exercise test and a PROM. Percent of predicted 6MWT had four statistically significant relationships with PROMs. Associations were very weak to weak, with *r*_s_’s ranging from −.18 (for school days missed) to .38 (for physical QOL).

The plank was the exercise test with the highest number of statistically significant relationships with the PROMs, namely seven. All associations were weak, with *r*_s_’s ranging from −.20 (for average pain) to .33 (for physical QOL).

The prone extension test had one statistically significant relationship with a PROM. It had a weak association with physical QOL (*r*_s_ = .26). More than half of the participants achieved a maximum prone extension test of 60 s, the median score.

The combined pushup measure had five statistically significant relationships with PROMs. All associations were weak, with *r*_s_’s ranging from .24 (for psychosocial QOL) to .32 (for physical QOL). Full body pushups and knee pushups each had three statistically significant, weak associations with PROMs, with *r*_s_’s ranging from .29 (for total QOL) to .33 (for physical QOL).

All statistically significant relationships between 6MWT, percent of predicted 6MWT, plank, prone extension and pushups combined were very weak to weak, except for the moderate relationship between plank and prone extension (*r*_s_ = .52). The correlation coefficients between different baseline PROMs are presented in Supplemental Table 1. Physical QOL is the only PROM with a statistically significant relationship to all seven exercise tests.

## Discussion

This study explored exercise testing results in a paediatric chronic pain population. Age stratification demonstrates minimal differences in exercise testing results between pre-adolescents and adolescents. The exception was the 6MWT and the percent of predicted 6MWT, whereby pre-adolescents walked further than adolescents (Table 2). This is contrary to norms data from a healthy population showing increased 6MWT distance as children age.^15^ It is difficult to know if this is due to different exercise capacities, or different motivation levels during exercise testing, for pre-adolescents and adolescents with chronic pain. Despite this difference, there were no statistically significant Spearman correlation coefficients between age and exercise testing results (Table 3).

This study also explored relationships between exercise testing results and self-reported baseline PROMs in a paediatric chronic pain population. Although one should be cautious in over-interpreting weak or null findings,^29^ the fact that exercise testing correlated only moderately at best with self-reported PROMS may imply that these measures are tapping into different but related constructs. Cognitive biases of youth with chronic pain,^30^ pain vigilance,^31^ difficulty with recall periods for different PROMs^32^ and a desire to address pain-stigma from parents and health professionals^33^ could account for the mostly very weak to weak associations between exercise testing and self-reported physical functioning. It is difficult to determine if cognitive development allows comparison of PROMs between children of different ages, although it is worth noting that this study used PROMs in validated, or at least published, age groups. This highlights the value of conducting a multi-pronged assessment, utilising exercise testing in addition to self-reported questionnaires.

Information from PROMs is thought to influence assessment and decision-making,^34^ improving communication between clinicians and patients to subsequently influence care.^35^ Similarly, the unique information from exercise testing may be used to help guide appropriate functional goal setting, as well as the development of graded exercise programs. Graded exercise programs could commence from the baseline level of activity, without causing a significant increase in symptoms.^36^

Very weak to moderate associations between different exercise tests suggests different information is obtained from the different tests, and of the exercise tests of interest, there was no standout. Pain assessments typically take more than one hour,^9^ so it is important for assessments to be meaningful and time efficient. A study exploring redundancy, or distinctness, of five different exercise tests concluded very weak to moderate correlation demonstrate enough difference to warrant performing each test.^37^ In our results, very weak to moderate correlation between different exercise tests indicate they each add different information to the assessment. Certainly, they each assess different muscle groups and forms of exercise capacity. However, the prone extension test had relatively high correlation to the plank test, and very low correlation with PROMs compared to the plank test, indicating possible redundancy of the prone extension test compared to the plank. This may have occurred due to a ceiling effect of a 60 s time limit for the prone extension test, whereby half this cohort achieved the full score. It is arguable that the 6MWT, plank and pushup tests are more worthwhile than the prone extension test.

### Limitations

The generalisability of the current findings is limited to paediatric patients, aged 6–18 years, attending a paediatric chronic pain service. One limitation of the retrospective study design was inconsistent reporting of why the physiotherapist deemed some participants did not complete exercise testing. Participants who refused an exercise test, or could not coordinate the test movement, were also not included in the data analysis. This limits the generalisability of study results to the more physically capable chronic pain cohort. The results are also not generalisable to non-English speaking or cognitively impaired patients as they could not complete PROMs for inclusion in the study. Another limitation due to the retrospective study design was the inability to analyse pain response and self-reported exertion during exercise testing. These were inconsistently recorded in electronic medical records and so were not analysed in this study. A benefit of our study taking place in a clinical setting is the generalisability of the assessment technique to other clinical settings. However, another limitation is posture during exercise tests may have been less-strictly controlled compared to a prospective study, affecting the reliability of the exercise tests.

Our 6MWT was completed on a 15 m track, compared to the recommended 30 m^14,15^ due to space limitations, confounding comparison to the reference equation. Nevertheless, it is worth noting that the 15 m track is validated and reliable,^38^ without substantially affecting the walk distance.^39^ The other exercise tests used in the current study have no accepted standard procedure, with variable protocols in published literature in healthy populations. For example, in an Australian norms dataset males and females completed pushups in 30 s^40^; whereas pushups were completed every three seconds in a reliability study.^26^ Although the exercise tests in our study were performed to a consistent protocol, the testing protocol varied from published literature. These changes, such as limiting the plank and prone extension test to 60 s, allows assessment of impairment in a pain cohort, without distinguishing between those with a higher exercise capacity. These restrictions also allow for more timely assessment in a busy clinical setting. Other changes, such as the arm position in the prone extension test, occur due to the longstanding personal preference of the assessor. It is difficult to determine the bias this introduces, and whether results are generalisable to similar (but not identical) exercise testing protocols.

The plank and pushup tests are validated in healthy populations from eight and nine years, respectively, not as young as our youngest participants.^24,26^ Only the 6MWT is validated in a chronic pain population, and not in a paediatric chronic pain population.^41^ This represents a wider gap in exercise testing literature. It is hard to know what bias the inclusion of six to eight year olds introduced, but it is reassuring that there were no obvious outliers in these younger age groups on dot plots (Supplemental Figure 1).

### Future research and clinical directions

A Delphi study may be useful to better understand how physiotherapists interpret subjective assessment, PROMs, exercise test results and/or pain response and exertion to formulate physical activity recommendations. Other exercise tests of interest could be identified, with only four distinct exercise types explored in this study. Ideally in the future gold standard exercise test procedures would be established. In the meantime, future research and clinical practice could prioritise exercise assessment procedures that allow for comparison to population norms, such as pushups in 30 s.^40^

Another avenue for future research in a paediatric chronic pain population is the role of exercise re-tests as an outcome measure, something not explored in this study. In adults with chronic pain, 6MWT was shown to improve following intervention, although this did not correlate to improvement in PROMs.^41^

The clinical meaningfulness of high or low correlation between exercise testing results and PROMs could be further explored. This could include if the low correlation is associated with poor self-awareness of the youth’s pain-impacted function. Future research could also determine the predictive capacity of comparing baseline exercise testing to PROMs for subsequent pain outcomes.

Future study designs that can include non-English speaking and cognitively impaired participants will improve understanding of exercise testing in these cohorts.

## Conclusions

Standardised exercise tests were presented and analysed in a paediatric chronic pain cohort, including with age stratification. There were minimal differences observed due to age, suggesting standardised tests can be applied to a paediatric pain population. There is very weak to moderate correlation between exercise testing results and a range of PROMs in a this cohort. This suggests exercise testing adds unique information to a multidisciplinary pain assessment, which could be used to guide treatment and recommendations. The 6MWT, plank and pushups show more utility than the prone extension test when considering correlation of exercise tests to PROMS, and other exercise tests. Multiple opportunities for future paediatric chronic pain research have been identified, with a priority to use standard exercise protocols with normative datasets for comparison. This allows results to be more comparable across settings.

## Supplemental material


Supplemental material – Exercise testing in a paediatric chronic pain cohort – A cross-sectional observation study
Supplemental material for Exercise testing in a paediatric chronic pain cohort – A cross-sectional observation study by Andrew Gorrie, Bethany McLennan, Emily Yeoh and Tiina Jaaniste in British Journal of Pain

Supplemental material – Exercise testing in a paediatric chronic pain cohort – A cross-sectional observation study
Supplemental material for Exercise testing in a paediatric chronic pain cohort – A cross-sectional observation study by Andrew Gorrie, Bethany McLennan, Emily Yeoh and Tiina Jaaniste in British Journal of Pain

## Data Availability

The authors confirm that the data supporting the findings of this study are available within the article and its supplementary materials. Raw data can be provided on reasonable request.*
