# Supplementary material for: Exercise testing in a paediatric chronic pain cohort – A cross-sectional observation study
Source: Br J Pain. 2026 Apr 29:20494637261445408. Online ahead of print. doi: 10.1177/20494637261445408 (PMC13128793; doi:10.1177/20494637261445408)
Supplement: Supplemental material – Exercise testing in a paediatric chronic pain cohort – A cross-sectional observation study [file sj-pdf-1-bjp-10.1177_20494637261445408.pdf]

Supplementary Table 1. Spearman Correlation Coefficients (and sample size) between Baseline PROMs and Baseline PROMs

	Pain - worst	Pain - average	QOL phys	QOL psycho social	QOL total	FDI	BAPQ	School Days Missed
Pain - average	.65[aa] (153)							
QOL phys	-.47[aa] (153)	-.44[aa] (153)						
QOL psycho social	-.23[a] (153)	-.20[a] (153)	.52[aa] (153)					
QOL total	-.37[aa] (153)	-.31[aa] (153)						
FDI	.50[aa] (149)	.42[aa] (149)	-.84[aa] (149)	-.58[aa] (149)	-.74[aa] (149)			
BAPQ	.19 (78)	.25[a] (78)	-.47[aa] (78)	-.66[aa] (78)	-.65[aa] (78)	.51[aa] (78)		
School Days Missed	.29[aa] (140)	.23[a] (140)	-.42[aa] (140)	-.23[a] (140)	-.34[aa] (140)	.44[aa] (137)	.13 (68)	
Age	.06 (153)	.03 (153)	-.07 (153)	-.06 (153)	-.08 (153)	.05 (149)	.03 (78)	.17[a] (140)

[a] P<.05, [aa] P<.001

QOL, quality of life; QOL phys, quality of life physical; FDI, Functional Disability Inventory; BAPQ, Bath Adolescent Pain Questionnaire Pain-Specific Anxiety Subscale; School Days Missed (in past fortnight)
