# Supplementary material for: Exercise testing in a paediatric chronic pain cohort – A cross-sectional observation study
Source: Br J Pain. 2026 Apr 29:20494637261445408. Online ahead of print. doi: 10.1177/20494637261445408 (PMC13128793; doi:10.1177/20494637261445408)
Supplement: Supplemental material – Exercise testing in a paediatric chronic pain cohort – A cross-sectional observation study [file sj-pdf-2-bjp-10.1177_20494637261445408.pdf]

Supplementary Figure 1. Baseline PROMs and Exercise Testing Results by Age Dot Plots

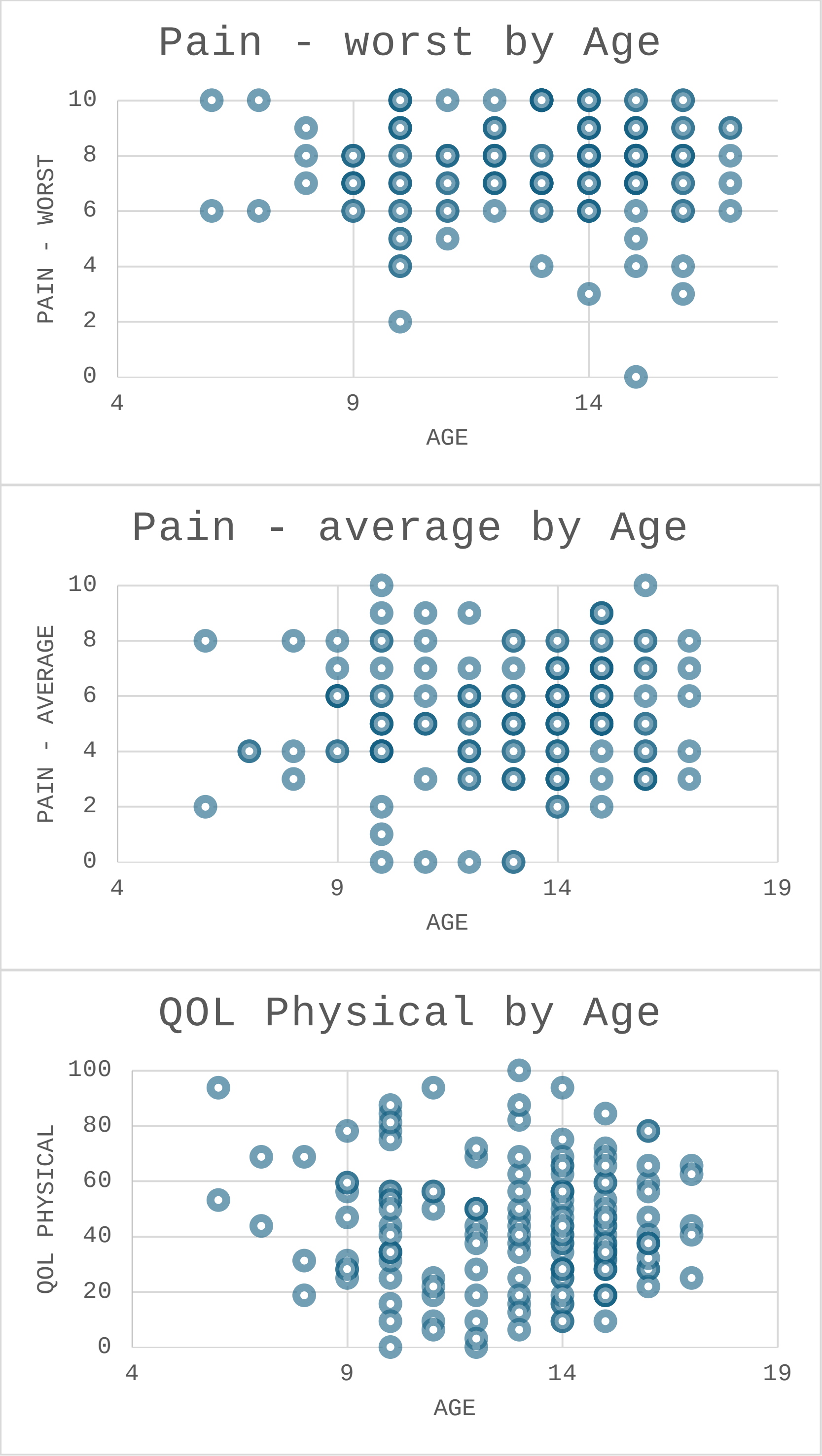

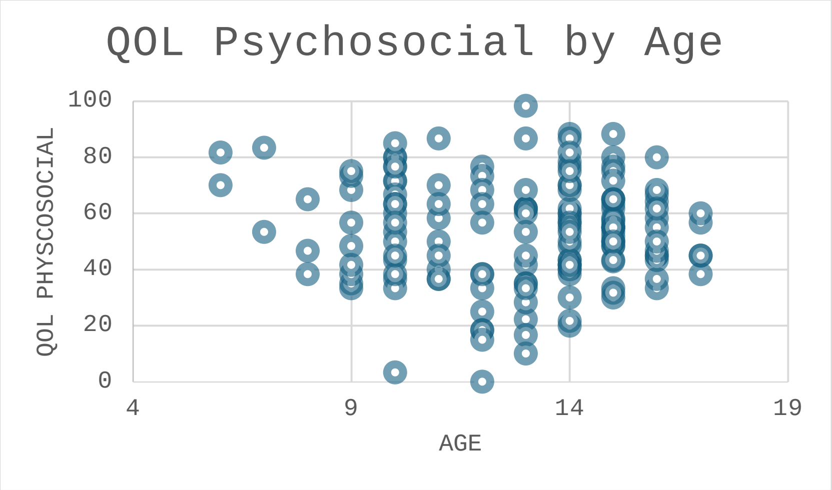
QOL Psychosocial by Age
QOL PHYSCOSOCIAL
100
80
60
40
20
0
4
9
14
19
AGE

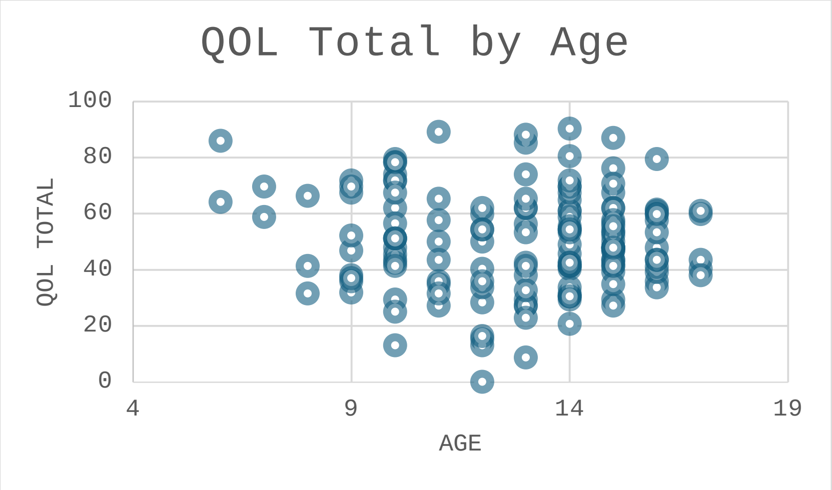
QOL Total by Age
QOL TOTAL
100
80
60
40
20
0
4
9
14
19
AGE

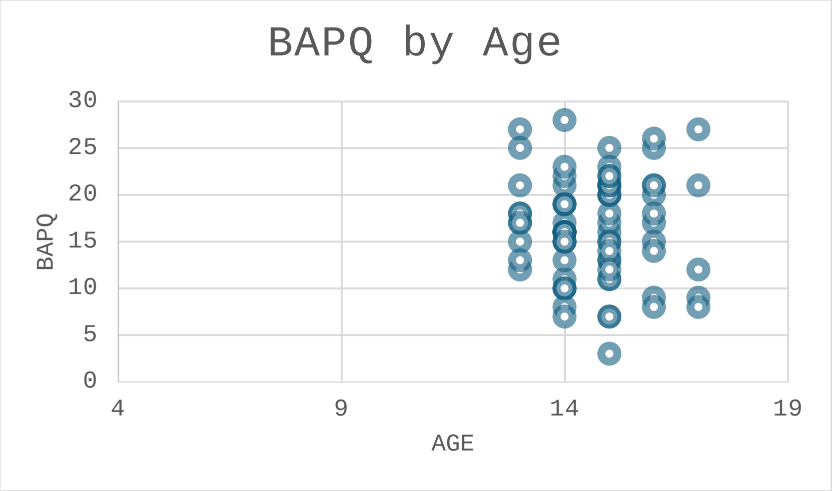
BAPQ by Age
BAPQ
30
25
20
15
10
5
0
4
9
14
19
AGE

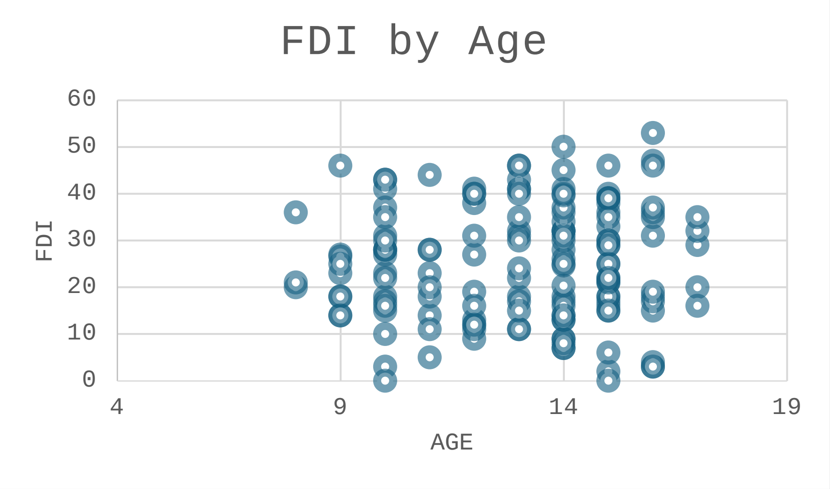

FDI by Age
FDI
60
50
40
30
20
10
0
4
9
14
19
AGE

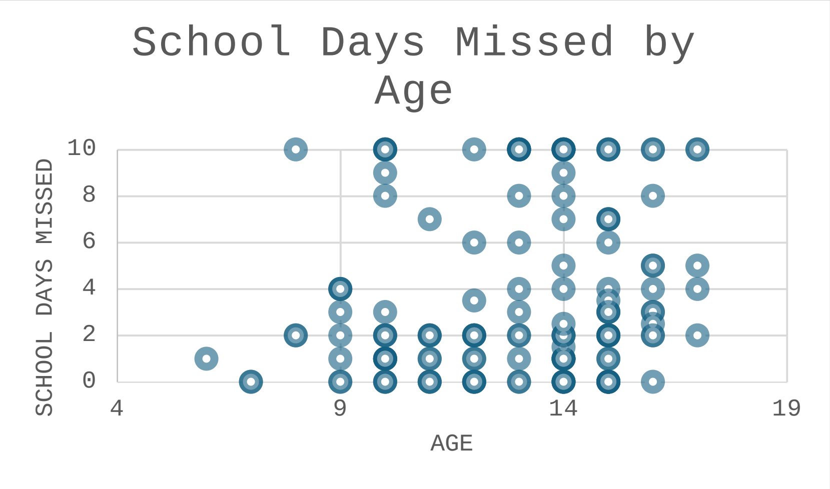

School Days Missed by Age
SCHOOL DAYS MISSED
10
8
6
4
2
0
4
9
14
19
AGE

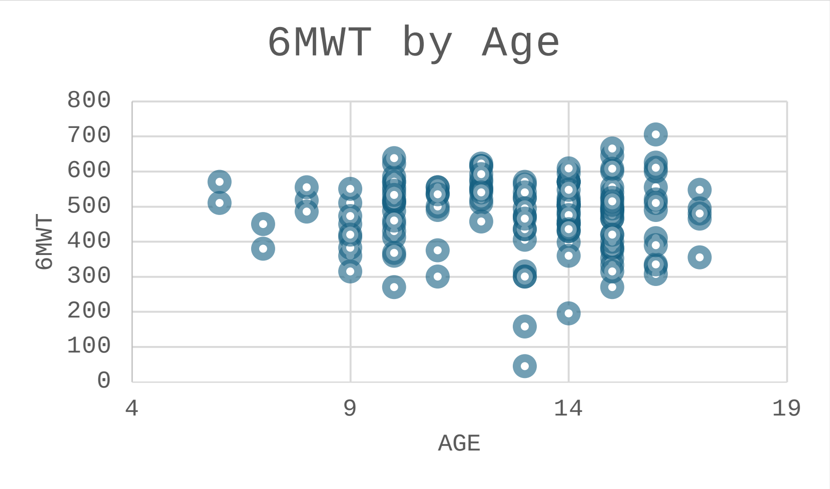

6MWT by Age
6MWT
800
700
600
500
400
300
200
100
0
4
9
14
19
AGE

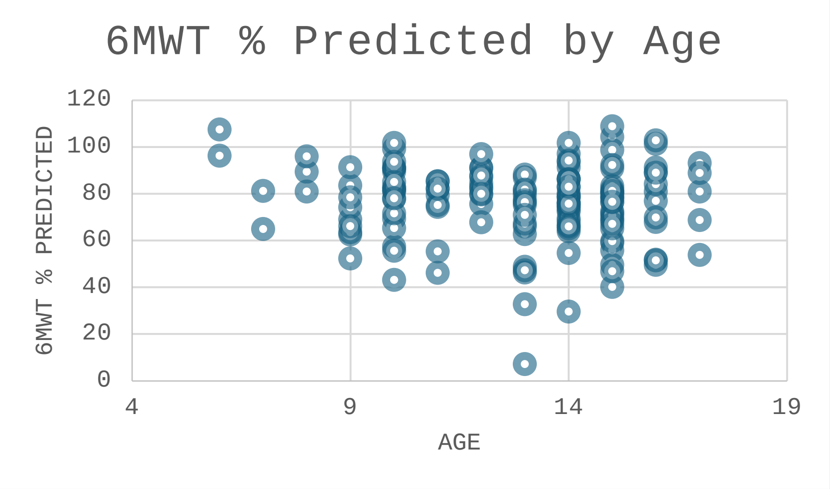

6MWT % Predicted by Age
6MWT % PREDICTED
120
100
80
60
40
20
0
4
9
14
19
AGE

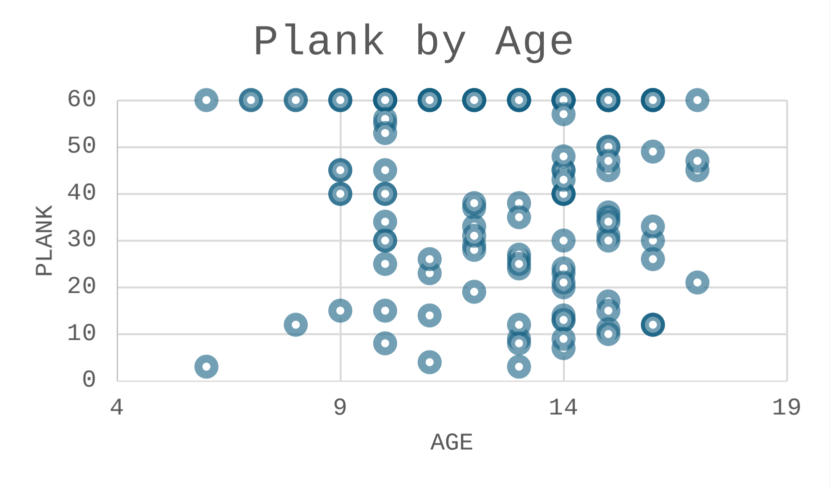

Plank by Age
PLANK
60
50
40
30
20
10
0
4
9
14
19
AGE

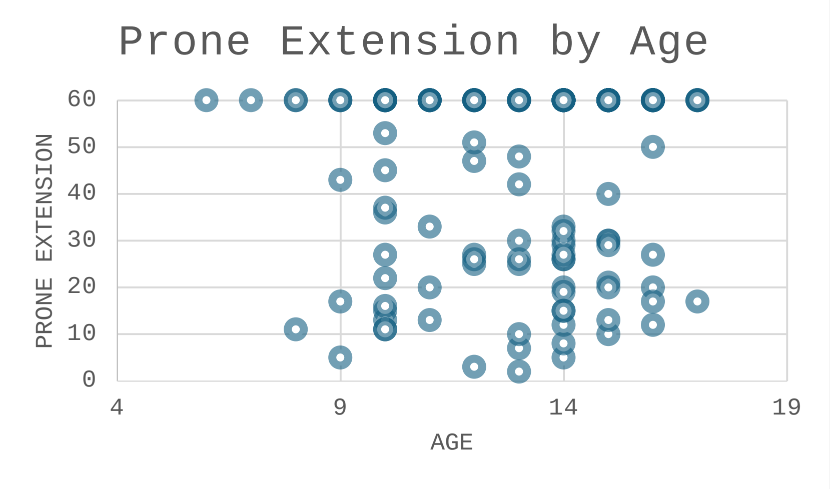

Prone Extension by Age
PRONE EXTENSION
60
50
40
30
20
10
0
4
9
14
19
AGE

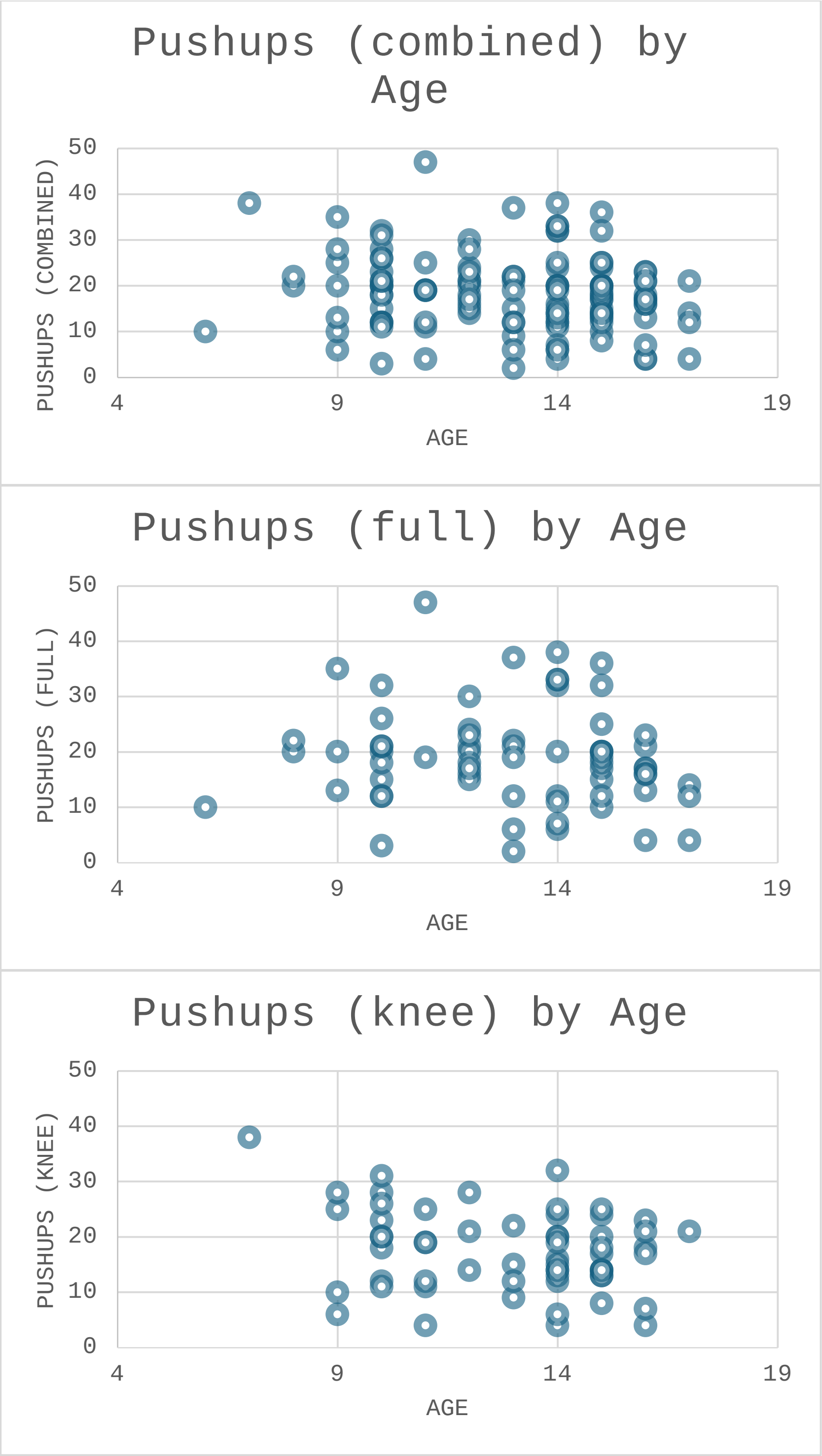

Figure S1. Scatterplot Correlation Between Physical QOL and 6MWT With Medians

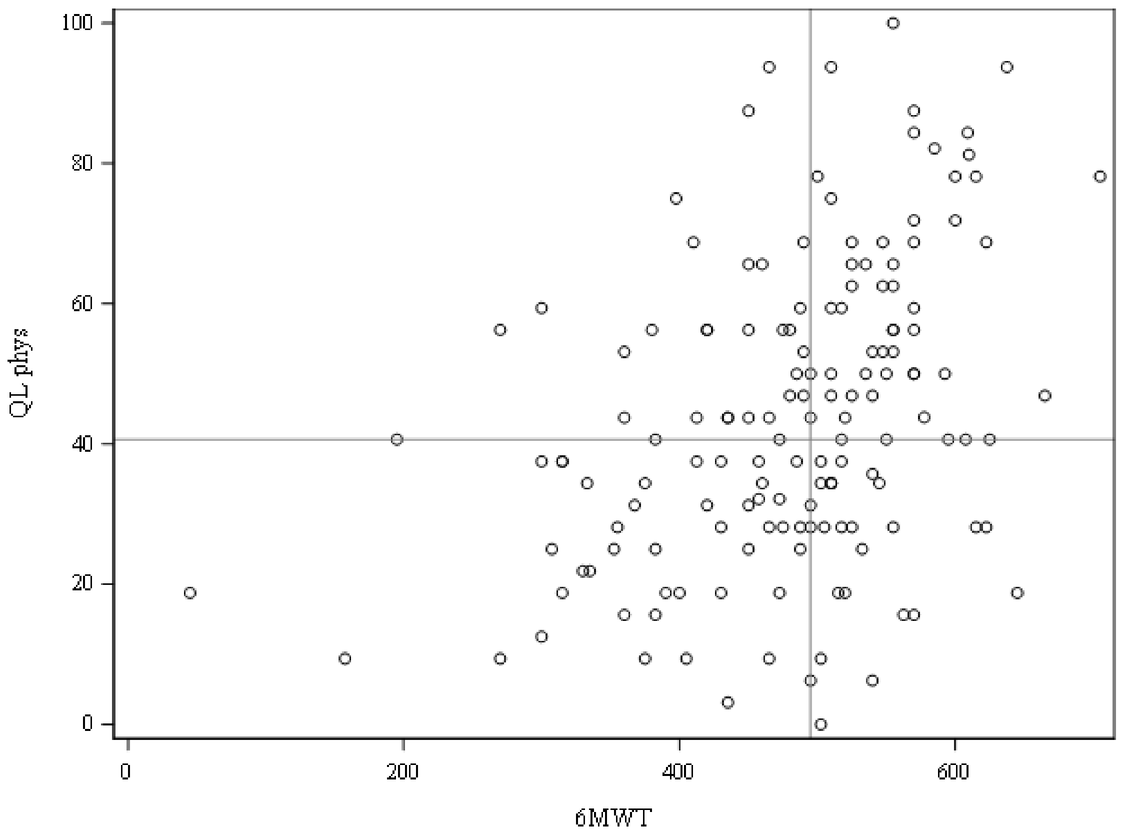

100
80
60
40
20
0
QL phys
0
200
400
600
6MWT
